# Altered white matter and cortical structure in neonates with antenatally diagnosed isolated ventriculomegaly

**DOI:** 10.1016/j.nicl.2016.01.012

**Published:** 2016-01-14

**Authors:** G. Lockwood Estrin, V. Kyriakopoulou, A. Makropoulos, G. Ball, L. Kuhendran, A. Chew, B. Hagberg, M. Martinez-Biarge, J. Allsop, M. Fox, S.J. Counsell, M.A. Rutherford

**Affiliations:** a Centre for the Developing Brain, Division of Imaging Sciences and Biomedical Engineering, King's College London, King's Health Partners, St. Thomas' Hospital, London SE1 7EH, United Kingdom; b Robert Steiner Unit, Imaging Sciences Department, MRC Clinical Sciences Centre, Hammersmith Hospital, Imperial College London, London W12 0HS, United Kingdom; c Gillberg Neuropsychiatry Centre, Institute of Neuroscience and Physiology, Sahlgrenska Academy at University of Gothenburg, Kungsgatan 12, 411 18 Gothenburg, Sweden

**Keywords:** Brain, Development, Ventricular enlargement, Magnetic resonance imaging, DTI, TBSS

## Abstract

Ventriculomegaly (VM) is the most common central nervous system abnormality diagnosed antenatally, and is associated with developmental delay in childhood. We tested the hypothesis that antenatally diagnosed isolated VM represents a biological marker for altered white matter (WM) and cortical grey matter (GM) development in neonates.

25 controls and 21 neonates with antenatally diagnosed isolated VM had magnetic resonance imaging at 41.97(± 2.94) and 45.34(± 2.14) weeks respectively. T_2_-weighted scans were segmented for volumetric analyses of the lateral ventricles, WM and cortical GM. Diffusion tensor imaging (DTI) measures were assessed using voxel-wise methods in WM and cortical GM; comparisons were made between cohorts.

Ventricular and cortical GM volumes were increased, and WM relative volume was reduced in the VM group. Regional decreases in fractional anisotropy (FA) and increases in mean diffusivity (MD) were demonstrated in WM of the VM group compared to controls. No differences in cortical DTI metrics were observed. At 2 years, neurodevelopmental delays, especially in language, were observed in 6/12 cases in the VM cohort.

WM alterations in isolated VM cases may be consistent with abnormal development of WM tracts involved in language and cognition. Alterations in WM FA and MD may represent neural correlates for later neurodevelopmental deficits.

## Highlights

• This study compared brain development in neonates with isolated VM to controls.
• Neonates with isolated VM have enlarged cortical volumes compared to controls.
• FA was reduced and MD was increased in the WM of the VM cohort.
• Children with antenatal isolated VM are at increased risk for language delay.

## 1 Introduction

Ventriculomegaly (VM) is the most common foetal central nervous system (CNS) abnormality diagnosed antenatally, affecting approximately 1% of foetuses. VM refers to the enlargement of the cerebral ventricles and is defined as an atrial diameter measurement of the lateral ventricle equal to or greater than 10 mm using antenatal ultrasound. VM is associated with abnormal development in childhood; with adverse neurodevelopmental outcome relating to both the severity of ventricular dilation, and the presence of associated abnormalities (Kazan-Tannus et al., 2007). Additional brain abnormalities are detected in approximately 50% of antenatally diagnosed VM cases. In the absence of other anomalies the term isolated VM is used but the aetiology of the dilation of these ventricles remains unknown (Kelly et al., 2001). However, there is evidence that long-term neurodevelopmental outcome is affected in cases of isolated VM (Melchiorre et al., 2009), with neurodevelopmental outcomes including difficulties in language (Falip et al., 2007, Sadan et al., 2007), cognitive (Bloom et al., 1997, Gomez-Arriaga et al., 2012, Leitner et al., 2009, Sadan et al., 2007), gross motor (Gomez-Arriaga et al., 2012, Leitner et al., 2009) and behavioural functions (Falip et al., 2007, Gomez-Arriaga et al., 2012, Leitner et al., 2009, Sadan et al., 2007).

In adults, an association has been observed between mild enlargement of the ventricles and neuropsychiatric disorders such as autism (Palmen et al., 2005), attention deficit hyperactivity disorder (Lyoo et al., 1996) and schizophrenia (Wright et al., 2000); although it is uncertain whether the observed VM in these adults had its origins in foetal life.

Antenatally diagnosed isolated VM has been hypothesised to be a structural marker for altered brain development. Brain alterations have manifested in increased total brain tissue in isolated VM compared to controls, which appears to be restricted to the cortex in foetuses (Kyriakopoulou et al., 2014) and neonates (Gilmore et al., 2008). Increased white as well as total grey matter (GM) volumes have also been observed in older children with antenatally diagnosed VM (Lyall et al., 2012). Diffusion tensor imaging (DTI) has demonstrated significant regional white matter (WM) changes in neonates born with isolated VM compared to controls (Gilmore et al., 2008, Goodlett et al., 2009), suggesting delayed or altered neurodevelopment.

Quantitative measurements derived from DTI can be used to make inferences about the underlying tissue structure (Beaulieu, 2002, Beaulieu and Allen, 1994, Pierpaoli et al., 1996), and has been used to demonstrate both white and cortical grey matter development in neonates (Ball et al., 2013b, Partridge et al., 2004). DTI is therefore an ideal technique to further understand microstructural alterations of WM and GM in infants with isolated VM. However to date there have been no studies using DTI to assess global WM or cortical GM development in a neonatal VM cohort.

Whole brain voxel-wise analyses, such as tract-based spatial statistics (TBSS), have the power to reveal DTI differences in WM tracts between groups of neonates (Anjari et al., 2009, Anjari et al., 2007, Ball et al., 2010), or to assess cortical microstructural maturation in preterm infants (Ball et al., 2013a). These approaches are ideal to objectively study global DTI differences between cohorts, allowing insight into the underlying WM and cortical GM structure of neonates with isolated VM compared to controls. This study aimed to assess WM and cortical GM structure in neonates with antenatally diagnosed isolated VM compared to controls. Both DTI and volumetric measures were used to test the hypothesis that isolated VM represents a biological marker for altered WM and cortical GM development.

## 2 Materials and methods

### 2.1 Subjects

Ethical approval and written parental informed consent was obtained prior to the scan for all participants (07/H0707/105, 07/H0707/101, 04/Q0406/125). Ethical approval was granted by the Northwest Hospitals Ethics Committee. A metal checklist was completed before the scan to ensure the infant was free of ferrous metals and safe to enter the magnetic field. All postnatal scans were performed in a dedicated 3-Tesla MR scanner located in the neonatal intensive care unit at the Queen Charlotte and Chelsea hospital.

#### 2.1.1 Normal controls

The normal control cohort consisted of 25 term-born infants from uncomplicated pregnancies. They comprised of 18 healthy volunteers, 3 infants with a sibling with a confirmed antenatal CNS abnormality not detected in the participating infant, 2 infants referred from the antenatal ultrasonography department for suspicion of a CNS abnormality which was excluded on MRI and 2 infants with a non-CNS abnormality which had resolved antenatally. All postnatal MR images of the normal control cohort were reviewed by an experienced perinatal neuroradiologist to confirm normal appearances for age. The following exclusion criteria applied: delivery complications with abnormal neurological signs, low birth weight (< 3rd centile), congenital malformations, signs of infection, chromosome abnormality, multiple pregnancy, premature delivery (< 36 weeks gestation), abnormal neonatal examination or neurodevelopmental outcome, and infants that had a non CNS-abnormality or had a sibling with an abnormality that did not have a formal developmental assessment.

#### 2.1.2 Isolated ventriculomegaly

The isolated ventriculomegaly cohort consisted of 21 infants with a diagnosis of antenatal isolated ventriculomegaly on MRI. Foetuses presenting with ventricular dilatation on antenatal ultrasound were referred to our department for a clinical antenatal MRI scan to further assess the developing brain. Atrial diameter was measured on ultrasound and MR images according to the International Society of Ultrasound in Obstetrics and Gynaecology (ISUOG) guidelines (ISUOG 2007), and ventriculomegaly was defined when the atrial diameter of one or both ventricles was ≥ 10 mm. All mothers attending for a clinical antenatal MRI were later invited for a postnatal MRI. Foetal MRI results of this cohort have previously been reported (Kyriakopoulou et al., 2014). Cases were only included in this neonate isolated VM cohort when there were no other brain abnormalities reported on the foetal or neonatal MRI, as reviewed by an experienced perinatal neuroradiologist. In addition, none of the cases included showed any signs of dysmorphic features or other congenital anomalies at the time of the post-natal scan. Exclusion criteria for isolated VM were: additional brain abnormalities on MRI, positive infection screen or chromosomal abnormality screening, maternal drug use, multiple pregnancies, intra-utero growth restriction, low birth weight (< 3rd centile). Not all foetuses underwent amniocentesis for genetic investigation and therefore delivery summaries were also reviewed to exclude any dysmorphic facial features or additional undiagnosed congenital malformations that could indicate an underlying genetic syndrome.

### 2.2 Neurodevelopmental assessment

Parents were invited for a detailed neurodevelopmental assessment of their child at 1 and 2 years. Assessments were performed by a clinical psychologist or paediatric neurologist. Griffiths Mental Development Scales (GMDS) was used at 1 year and Bayley Scales of Infant Development-III (BSID-III) assessment was chosen for the 2 year neurodevelopmental assessment. GMDS assesses locomotor, personal-social, hearing and speech, hand-eye co-ordination and performance. Sub-Quotients (SQ) and Developmental Quotients (DQ) were calculated from the raw scores for each domain; a DQ below 88 (1SD), and a SQ below 84 (1SD) indicated developmental delay. The BSID-III assesses cognition, language (expressive and receptive) and motor (fine and gross) (Bayley, 2006). Composite and scaled scores for each developmental domain can be compared across all 3 BSID-III scales. Composite scores are scaled to a metric with a range from 40–160, a mean of 100 and standard deviation of 15. Scaled scores range from 1–19 with a mean of 10 and a standard deviation of 3. Developmental delay was classified when composite scores were below 85 (1SD) or the scaled scores were below 7 (1SD).

In cases where parents were unable to attend a formal assessment, questionnaires were provided. Ages and Stages Questionnaires-III (ASQ-3) and PedQ are parent-completed developmental questionnaires that assess the child's development in the areas of communication, motor, problem solving and personal-social. The reliability and validity of both questionnaires have been demonstrated (Gollenberg et al., 2010, Varni et al., 2003, Woodward et al., 2011). ASQ-3 serves as a first-level screening system and can identify infants or young children (between 1 month and 5.5 years) who are delayed in their development. Comparatively, PedQ was designed to assess a paediatric population (5 to 7 years) for healthy outcome (Varni et al., 2003). PedQ was only used in control cases if the GMDS, BSID-III or ASQ-3 had not been completed before 5.5 years; in any cases of concern for the child's development, a paediatric neurologist followed up the case. Socioeconomic classification and affluence rating were extrapolated from parental home postcode at the time of the child's birth.

### 2.3 Neonatal scanning procedure

MR imaging was performed on a 3-Tesla Philips Achieva system sited on the neonatal intensive care unit, using an eight-channel phased array head coil. Single-shot echo planar DTI was acquired in 32 non-collinear directions with the following parameters: TR 8000 ms; TE 49 ms, voxel size 1.75 × 1.75 × 2mm^3^; b value 750 s/mm^2^; SENSE factor 2. T_2_-weighted fast spin echo images were acquired using: TR 9000 ms; TE 160 ms; flip angle 90°; slice thickness 2 mm with 1-mm overlap; voxel size 0.86 × 0.86 × 1 mm; SENSE factor 2.

All parents were offered the option of their child receiving sedation for the scan (oral chloral-hydrate, 30–50 mg/kg). Following written parental informed consent, all infants with antenatal VM and 6 infants from the normal control cohort received sedation. The remaining control infants were scanned during natural sleep after being fed and swaddled. All neonates were clinically assessed as stable prior to scanning by an experienced paediatrician neonatal heart rate, oxygen saturation and temperature were monitored throughout the scan. Ear protection during scanning comprised of neonatal earmuffs (Natus MiniMuffs; Natus Medical Inc., San Carlos, CA) as well as individually moulded earplugs using silicone-based dental putty (President Putty, Coltene/Whaledent, Mahwah, NJ), which were placed into the external ear. A neonatologist experienced in MRI procedures supervised all examinations. A perinatal neuroradiologist reviewed the images.

### 2.4 DTI analysis

All DTI data were reviewed during the examination for the presence of motion artefact. If artefacts were present, the acquisition was repeated. Images were then visually assessed after the scan on a slice by slice basis, and slices with artefact were excluded. In cases with excessive motion throughout the scan, the entire dataset was discarded from the analysis.

DTI data were processed offline using FMRIB's Diffusion Tool Box (FDTv2.0), part of FSL (Smith et al., 2006, Smith et al., 2004). Initially, DTI data were affine registered to the non-diffusion weighted (b_0_) image to minimise distortions due to eddy currents, and to correct for small subject movements occurring during the scan acquisition. Non-brain tissue was then removed using the FSL Brain Extraction Tool, and fractional anisotropy (FA) and mean diffusivity (MD) images were produced by fitting a tensor model to the raw diffusion data using FDT (FMRIB's Diffusion Toolbox).

#### 2.4.1 Tract based spatial statistics

TBSS (Smith et al., 2006, Smith et al., 2004) was performed using an optimised protocol for neonatal DTI analysis (Ball et al., 2010). A target FA map was chosen (the target was chosen to have the median age of the study group: 40.86 weeks) and each infant's FA map was aligned in the target space, and a mean FA map was created. A second set of registrations was then performed to register every individual FA map to the mean FA map. The aligned images were then used to create the final mean FA map and a mean FA skeleton, which represented the centre of all tracts common to the group. An FA threshold of ≥ 0.15 was applied to the skeleton, to include the major WM pathways but exclude peripheral tracts where there was significant variability between subjects and partial volume effects with GM or cerebral spinal fluid (CSF). Each subject's aligned FA and MD data were then projected onto the skeleton for statistical analysis. The registration results of each neonate were visually assessed by two experienced researchers to ensure accurate registration.

#### 2.4.2 Cortical analysis

Cortical diffusion data from each subject was aligned into a common space to allow voxel-wise statistical analysis of cortical FA and MD between groups, as described by (Ball et al., 2013a). Briefly, non-linear registration was initially used to align each subject's T_2_ image to population-based anatomical templates (Serag et al., 2012). After tissue segmentation (described below), subject-specific cortical maps were transformed in the template space alongside co-registered FA and MD maps. A mean cortical map was produced and skeletonised. For each subject, a perpendicular search was carried out to find voxels near to the skeleton with the highest probability of belonging to the cortex in each subject. FA and MD were projected from these voxels onto the cortical skeleton for statistical analysis.

#### 2.4.3 Statistical analysis: tract based spatial statistics and cortical analysis

Using FSL's Randomise tool (Winkler et al., 2014), a voxel-wise permutation-based analysis was used to compare FA and MD measures between the control and VM cohorts, correcting for post-menstrual age (PMA) at scan, gestational age (GA) at birth and sex. The results were corrected for multiple comparisons by controlling family-wise error rate following threshold-free cluster enhancement, with p < 0.05 considered significant.

### 2.5 Volumetric analysis

All T2-weighted images were reviewed during the scan for the presence of motion, image quality and brain coverage. If artefacts were present, the scan was repeated. In cases with excessive motion the T_2_-weighted images were registered and reconstructed according to (Jiang et al., 2007a).

Automated segmentation was conducted on each neonatal T_2_ scan in order to segment brain tissue and extract volume measures from the cortex, lateral ventricles and WM using a neonatal specific segmentation approach (Makropoulos et al., 2014) based on the Expectation–Maximisation (EM) technique (Van Leemput et al., 1999). Briefly, the neonatal brain was segmented by initially registering 20 manually segmented atlases (Gousias et al., 2012) to each individual's T_2_ image. After registering these atlases to individual T_2_ images, a spatial prior was generated for each region. The spatial priors were combined with information on the intensity of the image, which was modelled with a Gaussian Mixture Model (GMM), in order to obtain the structural segmentation of the brain. The influence of the intensity information in Makropoulos et al., (2014) was reduced in homogeneous areas where intensity-based delineation is less reliable. Markov Random Field (MRF) regularisation was utilised to enforce a smoothing labelling correcting for noisy or artefacted voxels. Partial volume correction was further performed at the boundary of cortical GM and CSF to correct for mis-labelled voxels.

To ensure accuracy of each segmentation, they were visually checked and manual editing performed with ITK-SNAP (Yushkevich et al., 2006). Volumetric measures of the cortex, lateral ventricles and WM of each neonate were extracted from each segmentation. The supratentorial tissue volume comprised of the sum of volumes from the cortex, WM and deep GM.

#### 2.5.1 Statistical analysis: volumetric analysis

Statistical analyses were performed using Stata/IC 11. Analysis of Covariance (ANCOVA) was performed to assess volumetric measurements between cohorts; PMA at scan and GA at birth were included as co-variables in the model.

## 3 Results

### 3.1 Subjects

The cohort characteristics of both groups can be seen in Table 1. There was no significant difference in GA at birth between cohorts. However, the PMA at scan in the isolated VM group was significantly greater than the control cohort (p < 0.001). Initial foetal MRI atrial diameter measurements of the lateral ventricles in the VM group ranged from of 10.5–17 mm (mean 12.5 mm). The cohort included 14 cases of unilateral ventriculomegaly, 1 of which was severe (17 mm) and 6 bilateral cases, of which 2 were severe (15.8 mm and 15.3 mm).

### 3.2 Neurodevelopmental assessments

20 control cases had neurodevelopmental assessments performed between 12.5 and 82 months (median 47 months), each with normal outcome. In further detail: a formal assessment at 2 years of age was conducted on 6 control children (mean age 25.6 months, range 20–29.4 months); one further case had a formal assessment at 11.7 months. 9 children had an ASQ-3 at a mean age of 4.3 years (range 3.25–5 years), and PedQ questionnaires were conducted on 4 control cases at a mean age of 6.42 years (range 6.08–6.83 years). The remaining cases were either too young for a formal assessment or lost to follow up.

A total of 17 isolated VM cases have had a formal neurodevelopmental follow up and no questionnaires were used. 12 isolated VM cases had a 2 year neurodevelopmental assessment (median age 24.8 (22.5–35) months); 6 of these cases had normal neurodevelopment and 6 displayed developmental delays. All 6 of these cases displayed delays in the language domain; 2 of these cases also demonstrated delays in cognitive and 1 case had a delay in gross motor, but not fine motor, skills. The cohort of 6 children that exhibited developmental delay had a mean foetal atrial diameter of 13.8 mm and range 10.7–17 mm. The cohort of 6 children that exhibited typical development had a mean foetal atrial diameter of 11.5 mm and range 10.5–12.5 mm. There was no significant difference in the socioeconomic classification distribution or the affluence rating between the cohort of children with developmental delay and those with typical development. A further 5 cases showed normal development at 1 year (11–13 months), however the assessment conducted at this age may be too early to detect language delays. The final 4 cases were lost to follow up; these cases were not different from those who had assessments in terms of delivery summary information, and all had been born in good condition with normal birth weight and their lateral ventricles had atrial diameter measurements of between 11.5–13 mm at their initial foetal MRI scan.

### 3.3 Tract based spatial statistics

**Fig. 1:**
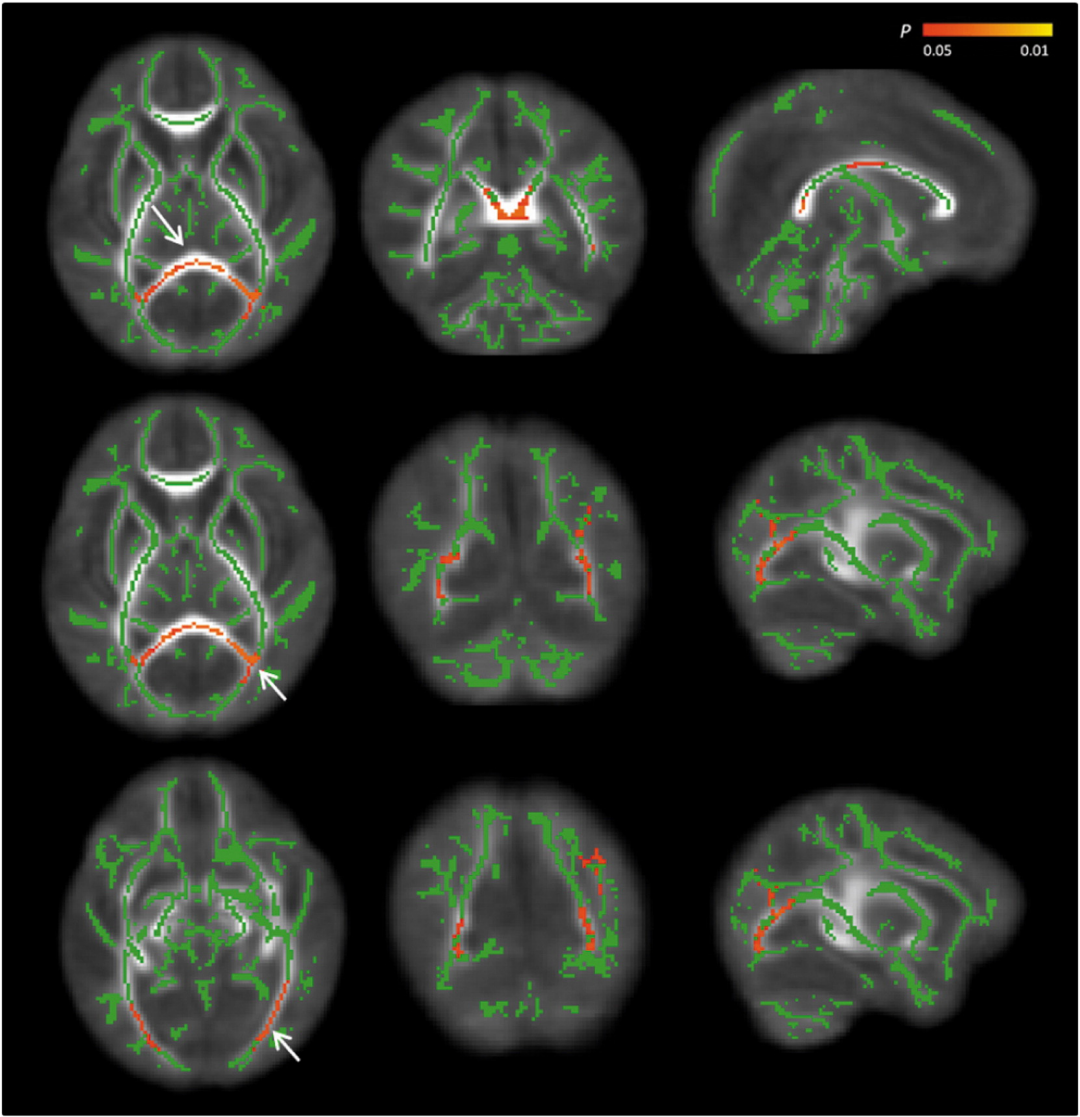
Differences in FA values between the 2 groups of infants. The mean FA skeleton (green) is overlaid on the mean FA map. Areas in red show regions where FA values were significantly lower in the VM group compared to controls (p < 0.05), after correcting for multiple comparisons following threshold-free cluster enhancement. Arrows demonstrate regions of FA reduction in the splenium (top row), posterior thalamic radiation (middle row) and sagittal stratum (bottom row) in the transverse, coronal and sagittal plane. (For interpretation of the references to colour in this figure legend, the reader is referred to the web version of this article.)

**Fig. 2:**
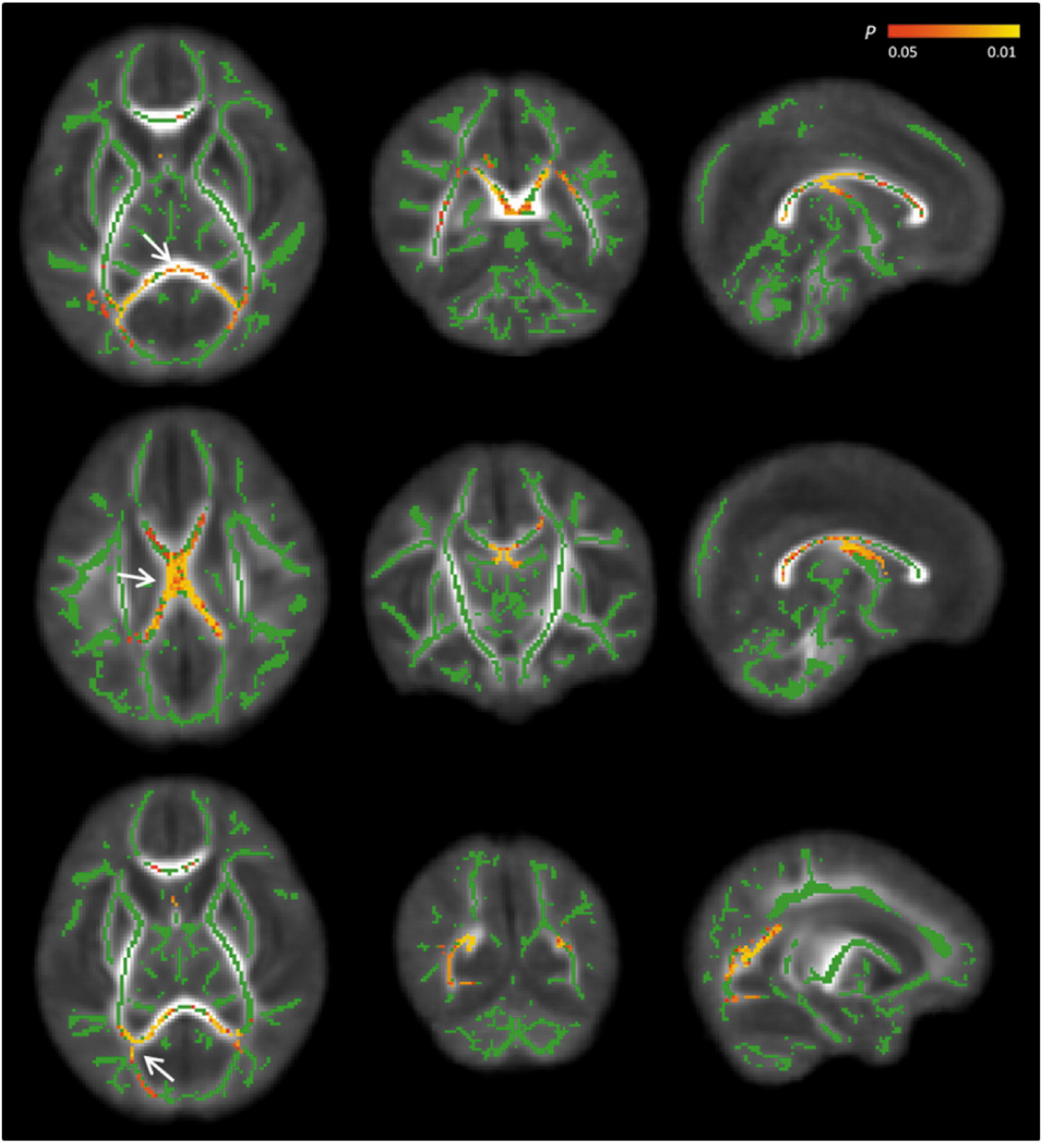
Differences in MD values between the 2 groups of infants. MD results are overlaid on the mean skeleton (green) and the mean FA map. Areas on the skeleton in red-yellow show regions where MD values were significantly increased in the VM group compared to controls. Arrows demonstrate regions of MD increase in the (top row) splenium and (middle row) body of the corpus callosum and fornix, and (bottom row) posterior thalamic radiation in the transverse, coronal and sagittal plane. (For interpretation of the references to colour in this figure legend, the reader is referred to the web version of this article.)

Significantly reduced FA values were found in the posterior thalamic radiation, sagittal stratum, splenium, and body of the corpus callosum (Fig. 1). MD values were increased in the posterior thalamic radiation, the splenium and body of the corpus callosum, and in the fornix in VM cases compared to controls (Fig. 2). There were no regions within the WM where FA values were higher or MD values were lower in the VM cohort compared to controls.

No significant differences in white matter FA or MD were found between males and females. When sex was included in the statistical model, the results between VM and control groups remained the same for white matter FA and MD.

### 3.4 Cortical analysis

From the original cohorts, 18 healthy controls and 17 neonates with antenatally diagnosed VM were included in the cortical GM analysis; other cases were excluded due to poor quality T_2_ data or mis-registration of DTI and T_2_ data. These sub-groups had a mean PMA at scan of 42.4 (± 3.29) and 45.3 (± 1.74) weeks, and a mean GA at birth of 39.2 (± 0.90) and 40 (± 1.26) weeks in the control and VM group respectively.

No significant FA or MD differences were found in DTI metrics in the cortex between the control and VM groups.

No significant differences in cortical FA or MD were found between males and females. When sex was included in the statistical model, there remained no significant differences in cortical FA or MD between the control and VM groups.

### 3.5 Volume analysis

**Fig. 3:**
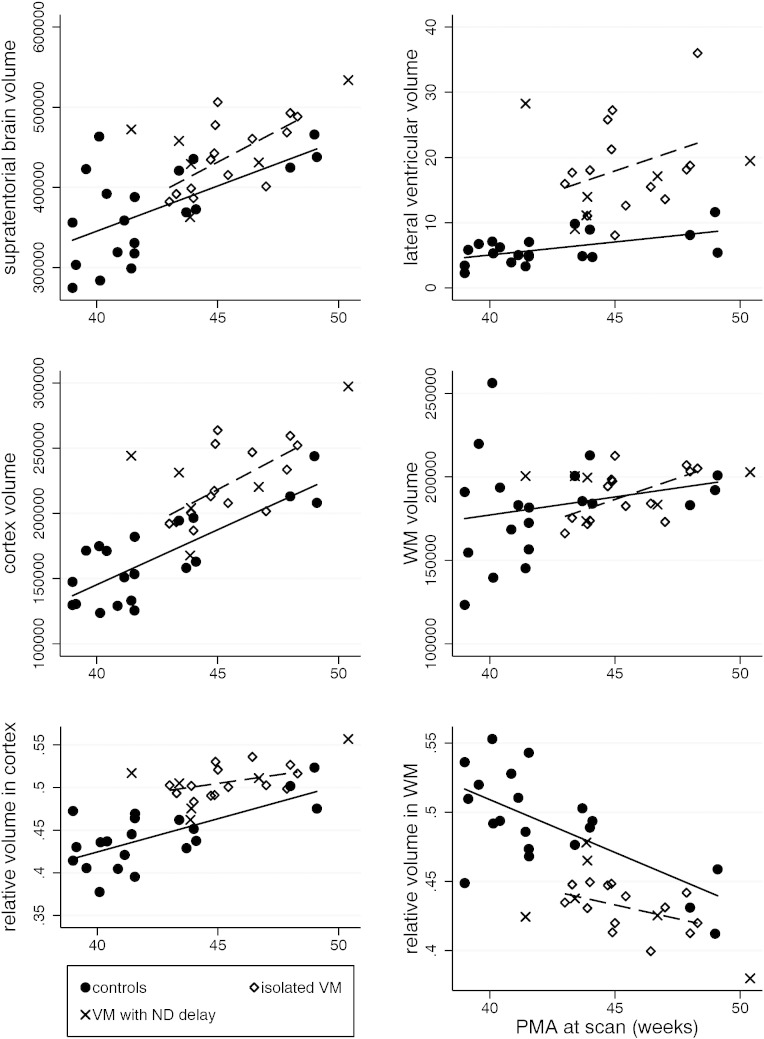
Absolute volume and relative volume measures in control and isolated VM cohorts. Absolute volume measures of the supratentorial tissue, lateral ventricles, cortex and WM are demonstrated in the control compared to isolated VM cohorts. Relative volume measures of the cortex and WM are shown in the control compared to isolated VM cohorts. Volume measures in mm^3^; ND = neurodevelopmental delay.

From the original cohorts, 20 healthy controls and 20 neonates with antenatally diagnosed VM were included in the volumetric analysis; other cases were excluded due to low quality T_2_ data. These sub-groups had a mean PMA at scan of 42.34 (± 3.17) and 45.32 (± 2.19) weeks, and a mean GA at birth of 39.40 (± 1.03) and 40.02 (± 1.24) weeks in the control and VM group respectively. The absolute volumes for each group can be seen in Table 2c. Fig. 3 shows the volume measures for both groups for each tissue type.

Supratentorial tissue volumes were significantly larger in the VM neonates compared to controls (p = 0.021; adj R^2^ = 0.520; b coefficient 39,186.7; 95% CI: 6184.3, 72,198.1).

Lateral ventricular volumes were significantly larger in the VM group compared to controls (p < 0.001; adj R^2^ = 0.570; b coefficient 10.69; 95% CI: 6.79, 14.58). This difference remained significant after controlling for total supratentorial tissue volume (p < 0.001; adj R^2^ = 0.602; b coefficient 9.23; 95% CI: 5.19, 13.27).

There was no significant difference in absolute WM volume between isolated VM and control cohorts (p = 0.763; adj R^2^ = 0.033). However, when total supratentorial brain tissue volume was controlled for, WM volume was significantly reduced in the isolated VM group compared to controls (p < 0.001; adj R^2^ = 0.808; b coefficient − 15,883.2; 95% CI: − 24,202.1, − 7564.3).

Cortical GM volume was significantly greater in the isolated VM group compared to controls (p < 0.001; adj R^2^ = 0.721; b coefficient 36,053.7; 95% CI: 18,659.6, 53,447.8). This difference remained significant after controlling for total supratentorial tissue volume (p < 0.001; adj R^2^ = 0.944; b coefficient 17,538.2; 95% CI: 9107.3, 25,969.2). There was no significant difference between genders in the volume of the lateral ventricles, white matter, supratentorial brain tissue or cortical volume in either the normal controls or ventriculomegaly cohorts.

## 4 Discussion

This study demonstrates altered WM and cortical GM development in infants with antenatally diagnosed isolated VM. These results provide evidence to support the hypothesis that isolated VM is a marker for altered brain development.

### 4.1 White matter alterations

We observed a regional reduction in FA and increase in MD values in the WM of neonates with isolated VM compared to controls; this was the first study to use an objective whole-brain approach to assess DTI measures in WM tracts of infants with isolated VM. The observed DTI differences, in addition to the reduction in WM relative volume, suggests altered WM structure in neonates with antenatally diagnosed isolated VM at term age.

Increased MD values in infants with VM may be caused by an increase in water content and a decrease in restriction to water motion, and as MD values typically decrease with increasing age (Bui et al., 2006, Partridge et al., 2004), increased regional MD values in neonates with isolated VM may represent delayed or altered WM maturation.

DTI measures of anisotropy are influenced by a number of microstructural barriers, including axonal membranes, coherent fibre organisation, degree of axonal packing and myelin (Beaulieu, 2009, Budde et al., 2008, Song et al., 2003, Takahashi et al., 2002). At term, normal myelination has only progressed from the brainstem to the PLIC (Yakovlev and Lecours, 1967), and so the observed regional WM FA alterations are likely illustrative of a disruption in the processes that occur leading up to myelination, such as increased axonal thickness, alteration in axonal permeability and pre-myelination wrapping of oligodendrocytes around axons (Wimberger et al., 1995).

Previous papers have also demonstrated similar decreases in FA and increases in MD in neonates with isolated VM compared to controls (Gilmore et al., 2008, Goodlett et al., 2009), but these studies relied upon DTI analysis in user predetermined WM regions. TBSS offers the advantage of objectively analysing whole-brain DTI data on a voxel-wise basis, and does not rely on a priori selection of regions. Gilmore et al. (2008) used a region of interest approach to assess neonates with isolated VM compared to controls and demonstrated a significant decrease in FA and increase in MD in the splenium and cortico-spinal tracts, as well as an increase in MD in the genu compared to controls. This work was extended by using a population-based registration method to compare FA values between groups; reduced FA values in the splenium of neonates with isolated VM were found, but not in the genu or corticospinal tracts (Goodlett et al., 2009). Our findings are consistent with these previous results, and we found additional FA decreases and MD increases in the posterior thalamic radiation and body of the corpus callosum in the VM group compared to controls; further FA reductions were also seen in the sagittal stratum, and MD increases in the fornix.

The corpus callosum, posterior thalamic radiation and sagittal stratum contain a number of WM tracts (Oishi et al., 2010, Wakana et al., 2004); fibres passing through the splenium of the corpus callosum form the forceps major. Both the posterior thalamic radiation and sagittal stratum contain fibres of the inferior and superior longitudinal fasciculus as well as the inferior fronto-occipital fasciculus. In addition, the posterior thalamic radiation contains the optic radiations. Injury to, or aberrant development of these WM tracts, specifically the superior and inferior longitudinal fasiculi, inferior fronto-occipital fasciculus, posterior thalamic radiations and the corpus callosum, are associated with deficits in language, motor, cognitive and attention skills (Catani, 2007, Doricchi and Tomaiuolo, 2003, Dramsdahl et al., 2012, Duffau et al., 2002, Gazzaniga, 2000, Hynd et al., 1995, Leclercq et al., 2010, Nosarti et al., 2004, Qiu et al., 2011, Tanabe et al., 1987, van Kooij et al., 2012). In particular, altered size and shape of the corpus callosum has been associated with developmental language disorders in children (Preis et al., 2000). Altered development in these key WM tracts may help explain deficits in the domains of language, motor, cognitive and attention skills have been observed in children with isolated VM (Gomez-Arriaga et al., 2012, Lyall et al., 2012, Sadan et al., 2007).

In this study, one half of the children with VM who completed a neurodevelopmental assessment at 2 years demonstrated neurodevelopment delay. Previous reports of the risk of neurodevelopmental delay in isolated VM cohorts have been highly variable, which may be due to non-standardised developmental assessment examinations, evaluation at difference age ranges and limited sample sizes. Despite this variability, previous studies have demonstrated that developmental delays in children with isolated VM predominantly includes delays in expressive and receptive language performance (Falip et al., 2007, Sadan et al., 2007), cognitive (Bloom et al., 1997, Gomez-Arriaga et al., 2012, Leitner et al., 2009, Sadan et al., 2007) and behavioural deficits (Falip et al., 2007, Gomez-Arriaga et al., 2012, Leitner et al., 2009, Sadan et al., 2007); fewer papers have also noted motor deficits in this cohort (Bloom et al., 1997, Gomez-Arriaga et al., 2012). These findings are consistent with our findings of predominantly language deficits, and to a lesser extent cognitive and gross motor delays, in the VM cohort.

The findings of alterations in DTI metrics in specific WM tracts associated with neurodevelopmental delays supports the hypothesis that isolated VM is a structural marker of altered brain development that may be associated with high risk for neurodevelopmental disorders. Future larger longitudinal studies which include neurodevelopmental follow-up are necessary to determine whether the regional WM changes in isolated VM neonates observed in this study are associated with subsequent outcome and whether DTI measures may therefore be used as an early marker of delayed neurodevelopment.

### 4.2 Cortical alterations

Cortical GM volumes were significantly larger in neonates with isolated VM compared to controls. This result is consistent with a number of studies that have shown cortical enlargement in foetuses (Kyriakopoulou et al., 2014), neonates (Gilmore et al., 2008) and children (Lyall et al., 2012), as well as with findings in a foetal rat model where VM was associated with cortical overgrowth (Eyles et al., 2003).

A recent in utero MRI paper from our group, using motion tolerant imaging techniques specifically developed for foetal MRI (Jiang et al., 2007b), demonstrated increased cortical GM volumes in foetuses with antenatal isolated VM compared to controls (Kyriakopoulou et al., 2014). Previous foetal MRI studies did not identify any significant differences in cortical GM volume in isolated VM cases (Grossman et al., 2006, Kazan-Tannus et al., 2007, Pier et al., 2011, Scott et al., 2013), but the majority of these studies did not use motion tolerant imaging techniques. However, one study identified delayed cortical gyrification in foetuses with isolated VM (Scott et al., 2013), which is consistent with the hypothesis of abnormal cortical maturation in this group.

The cohort of foetuses with isolated VM studied by Kyriakopoulou et al., (2014) were followed up and had a neonatal MRI, the results of which are reported in this paper, and provides evidence that antenatally diagnosed isolated VM is associated with enlarged cortical volumes that remain enlarged through to infancy. Our results are consistent with a previous study of 34 neonates with antenatally diagnosed isolated VM compared to 34 aged-matched and sex-matched term controls, which found cortical volumes to be 10.9% greater in the isolated VM cohort (Gilmore et al., 2008); this study also found no difference in absolute WM volumes, but when controlling for intracranial volume, they showed smaller WM volumes with larger intracranial volumes. This volume alteration appears to persist into childhood, with enlargement of the lateral ventricles being associated with increased WM and total GM volumes at 2 years (Lyall et al., 2012). It has been suggested that cortical enlargement in antenatal isolated VM results from a lack of normal developmental apoptosis; apoptosis is prominent in the cortex from approximately 32 weeks gestation (Kuan et al., 2000). An increase in proliferating cells has also been implicated (Eyles et al., 2003), as animal studies have found a correlation between ventricle size and the amount of neuronal cell proliferation within the corresponding periventricular region (Sawamoto et al., 2006).

DTI has previously been used to investigate cortical maturation in preterm neonates, and has demonstrated a decline in cortical FA values across gestation (Ball et al., 2013b, Deipolyi et al., 2005, McKinstry et al., 2002); this FA reduction has been suggested to reflect neurite outgrowth and maturing dendritic cytoarchitecture which transform the cortex from a predominantly radial formation into a denser, more complex structure during this time (Bystron et al., 2008). These processes restrict water motion both orthogonally and radially to the cortical surface which may explain the reduction in anisotropy. Considering the delayed cortical development observed in brain volume analyses of foetuses and neonates with isolated VM compared to controls, we hypothesised the normal FA reduction observed with increasing gestation would also be delayed in an isolated VM cohort. However, despite volumetric results suggesting abnormal cortical development in neonates with isolated VM, no significant differences in the structure of the cortex, as measured by FA or MD, was found between the two groups. It is possible that the increase in cortical volume associated with antenatal isolated VM does represent an increase in cell numbers or synaptic connectivity due to disruption in the regulation of cell proliferation or apoptosis, as previously hypothesised (Gilmore et al., 2008, Kyriakopoulou et al., 2014). Our ability to identify subtle microstructural alterations in cortical GM is limited using DTI approaches. New imaging tools, such as neurite orientation dispersion and density imaging (NODDI) (Zhang et al., 2012) may provide greater understanding on the underlying microstructure associated with cortical overgrowth in infants with isolated VM.

## 5 Limitations

There were a number of limitations with the current study. Whilst voxel-wise approaches have an advantage over other ROI approaches, TBSS is limited to the centre of WM tracts, and any alterations in peripheral WM tracts would not be recognised.

In addition, our sample sizes were relatively small, especially for the cortical and volumetric analysis where numbers were reduced due to some cases having poor quality T_2_ data. The rate of developmental delay reported in our study is higher compared with the current literature and this may be due to the small size of our cohort and the fact that parents with concerns regarding their child's developmental progress may be more inclined to attend a developmental assessment. It is also possible that this reported high rate of developmental delay reflects the VM severity of individuals that were included in the follow up, as more severe VM is associated with poorer outcomes (Ouahba et al., 2006).

There was also a significant difference in PMA at scan between the two cohorts; however, this difference was taken into consideration during statistical analysis. The reason for this age difference was due to the recruitment protocol for control cases compared to clinical cases. The VM cohort also contained a greater number of male participants, and we therefore controlled for sex in the analysis. No significant DTI or volume differences were found between males and females. Previous neonatal DTI papers (Aeby et al., 2012, Alexandrou et al., 2014, Anjari et al., 2007, Ball et al., 2010) and unpublished results from a neonatal study in our department have also shown no significant DTI differences between male and female neonates.

Another limitation is that genetic investigations were not performed in all cases. Genetic investigation was only completed when parents agreed antenatally or when clinical signs in neonates suggested a potential genetic abnormality. Therefore, it is possible that more subtle genetic abnormalities might be present in some of the apparently isolated VM cohort. However, delivery summaries were reviewed to exclude any obvious features that could indicate an underlying genetic syndrome. Finally, we were unable to conduct a 2 year neurodevelopmental assessment on all control and VM cases; although these cases did not appear to have any differences compared to other individuals within the cohort with respect to delivery details. Neurodevelopmental assessments were completed at an age range of 11–82 months, and due to this large age range and 4 different assessments used, it was not possible to establish if there was a relationship between performance and the MR measures. However, future investigation with this VM cohort aims to assess development at 4 years using the Autism Diagnostic Observation Schedule (ADOS-2); and with this data it will be possible to establish whether DTI and volume measures are associated with longer term developmental outcome.

Larger cohorts in future investigations would also be useful to enable sub-group analyses of neonates with mild, moderate and severe isolated VM, in order to investigate whether brain development differs between these groups and controls.

## 6 Conclusions

Neonates with isolated VM displayed significantly enlarged cortical volumes compared to controls, but there was no alteration in the structure of the cortex as measured by FA or MD. TBSS demonstrated reduced FA and increased MD values in WM tracts in neonates with antenatally diagnosed VM. Antenatally diagnosed isolated VM cases appeared to have an increased risk of neurodevelopmental deficits, especially in the language domain. The observed DTI WM alterations in isolated VM cases may be consistent with a delay in maturation or abnormal development of specific WM tracts that are involved in language, cognition and motor skills. FA and MD alterations may therefore represent neural correlates for later neurodevelopmental deficits.

## Funding

The authors acknowledge financial support from the Medical Research Council (UK), and the Department of Health via the National Institute for Health Research (NIHR) (MRXBADR) comprehensive Biomedical Research Centre award to Guy's & St Thomas' NHS Foundation Trust in partnership with King's College London and King's College Hospital NHS Foundation Trust.

## Figures and Tables

**Table 1:** Cohort characteristics.

Cohort details	Normal control	Isolated VM
Gestational age at birth (weeks)	39.59 (± 1.06)	39.82 (± 1.50)
Post-menstrual age at scan (weeks)	41.97 (± 2.94)	45.34 (± 2.14)
Male	13	15
Female	12	6
Birth weight (kg)	3.36 (± 0.47)	3.45 (± 0.49)
Apgar score at 1 min	9 (8–10)	9 (7–10)^a^
Apgar score at 5 min	10 (9–10)	10 (10–10)^a^

GA at birth and PMA at scan are presented average (SD); Apgar scores are presented as median (range).

a Apgar scores were not available in 4 VM neonates, but delivery summaries for these cases noted that they had been born in good condition.

**Table 2:** Absolute Volume Measures.

Tissue segmentation	Absolute volumes (mm^3^)	
Control cohort	Isolated VM cohort
Supratentorial tissue	371,809.2 (± 60,326.92)	441,758.7 (± 46,326.29)
Lateral ventricles	5.97 (± 2.31)	17.93 (± 7.03)
White matter	182,244.6 (± 30,085.47)	190,279.2 (± 14,325.85)
Cortex	165,047.8 (± 33,577.03)	224,337.1 (± 31,915.42)
